# The P2X_7_ Receptor in Microglial Cells Modulates the Endolysosomal Axis, Autophagy, and Phagocytosis

**DOI:** 10.3389/fncel.2021.645244

**Published:** 2021-03-15

**Authors:** Keith E. Campagno, Claire H. Mitchell

**Affiliations:** ^1^Department of Basic and Translational Science, University of Pennsylvania, Philadelphia, PA, United States; ^2^Department of Ophthalmology, University of Pennsylvania, Philadelphia, PA, United States; ^3^Department of Physiology, University of Pennsylvania, Philadelphia, PA, United States

**Keywords:** P2X_7_, microglia, phagocytosis, autophagy, lysosomes, cathepsin B, NLRP3, neuroinflammation

## Abstract

Microglial cells regulate neural homeostasis by coordinating both immune responses and clearance of debris, and the P2X_7_ receptor for extracellular ATP plays a central role in both functions. The P2X_7_ receptor is primarily known in microglial cells for its immune signaling and NLRP3 inflammasome activation. However, the receptor also affects the clearance of extracellular and intracellular debris through modifications of lysosomal function, phagocytosis, and autophagy. In the absence of an agonist, the P2X_7_ receptor acts as a scavenger receptor to phagocytose material. Transient receptor stimulation induces autophagy and increases LC3-II levels, likely through calcium-dependent phosphorylation of AMPK, and activates microglia to an M1 or mixed M1/M2 state. We show an increased expression of *Nos2* and *Tnfa* and a decreased expression of *Chil3* (*YM1*) from primary cultures of brain microglia exposed to high levels of ATP. Sustained stimulation can reduce lysosomal function in microglia by increasing lysosomal pH and slowing autophagosome-lysosome fusion. P2X_7_ receptor stimulation can also cause lysosomal leakage, and the subsequent rise in cytoplasmic cathepsin B activates the NLRP3 inflammasome leading to caspase-1 cleavage and IL-1β maturation and release. Support for P2X_7_ receptor activation of the inflammasome following lysosomal leakage comes from data on primary microglia showing IL-1β release following receptor stimulation is inhibited by cathepsin B blocker CA-074. This pathway bridges endolysosomal and inflammatory roles and may provide a key mechanism for the increased inflammation found in age-dependent neurodegenerations characterized by excessive lysosomal accumulations. Regardless of whether the inflammasome is activated *via* this lysosomal leakage or the better-known K^+^-efflux pathway, the inflammatory impact of P2X_7_ receptor stimulation is balanced between the autophagic reduction of inflammasome components and their increase following P2X_7_-mediated priming. In summary, the P2X_7_ receptor modulates clearance of extracellular debris by microglial cells and mediates lysosomal damage that can activate the NLRP3 inflammasome. A better understanding of how the P2X_7_ receptor alters phagocytosis, lysosomal health, inflammation, and autophagy can lead to therapies that balance the inflammatory and clearance roles of microglial cells.

## Introduction

Microglia act as the innate immune effector cells of the central nervous system (CNS; Gehrmann et al., 1995; Kofler and Wiley, 2011), and their contribution to inflammatory signaling pathways between neurons, astrocytes, and other components of neural tissue largely sets the magnitude of the immune responses. Although microglia constitute a relatively small number of cells in the CNS (Mittelbronn et al., 2001), their position as the primary defensive cells in neural systems places them at the crossroads of health and disease. The inflammatory responses mediated by microglial cells are regulated by multiple stimuli that help ensure an appropriate response. Extracellular ATP acting at the P2X_7_ receptor is widely recognized as a pivotal player in microglial signaling for triggering the release of neurotrophic factors, cytokines such as IL-6, and the activation of the NOD-, LRR- and pyrin domain-containing protein 3 (NLRP3) inflammasome (Kierdorf and Prinz, 2017).

In addition to their role in inflammatory signaling, microglial cells make major contributions to the clearance of material from extracellular space. Their ability to phagocytose and degrade apoptotic cells, bacteria, and engulfed synaptic material contributes to neural homeostasis (Nau et al., 2014; Bilimoria and Stevens, 2015). Furthermore, the dysfunctional clearance of proteins and lipids by microglia is increasingly implicated in neurodegenerative disorders. For example, undegraded myelin and lipid droplets have been observed within microglia from aging models (Safaiyan et al., 2016; Marschallinger et al., 2020). The incomplete removal of amyloid-beta (Aβ) deposits contributes to waste accumulation and “microglial exhaustion” in pathologies such as Alzheimer’s disease (Streit et al., 2018, 2020). These observations suggest that defective clearance of waste material by microglia may contribute to the mechanisms underlying some age-related neurodegenerations.

The multiple steps required for the phagocytosis and degradation of extracellular waste material by microglial cells are tightly coordinated. Several lines of evidence suggest the P2X_7_ receptor is involved in regulating microglial clearance, including effects on phagocytosis, autophagy, and lysosomal function. This review examines the evidence for the role of the P2X_7_ receptor in regulating these steps and highlights the role of the P2X_7_ receptor in modulating both the clearance and inflammatory components of microglial function.

## Extracellular Atp and P2x_7_ Receptors

Purinergic signaling pathways evolved early, and the ubiquitous presence of high cytoplasmic adenosine triphosphate (ATP) levels, combined with multiple pathways for ATP release, ensured widespread use of ATP as an extracellular transmitter. ATP can be released physiologically through ion channels or from vesicles loaded with ATP (Beckel et al., 2018; Dosch et al., 2018). Increased extracellular ATP is often associated with pathological stress; this rise in extracellular ATP concentration can result from upregulation of channel and vesicular release pathways, in addition to the release of cytoplasmic ATP upon cell rupture. Signaling is largely mediated by stimulation of ionotropic P2X or metabotropic P2Y receptors by ATP, the dephosphorylated nucleotide adenosine diphosphate (ADP), or pyrimidine analogs (Calovi et al., 2019). Adenosine produced by the subsequent dephosphorylation can act at a distinct set of metabotropic receptors, adding to the complexity. This also emphasizes the role of extracellular dephosphorylating enzymes in coordinating purinergic signals.

While most cell types possess some combination of purinergic receptors, purinergic signaling plays a particularly important role in microglial cells. The P2Y_12_ receptor for ADP is widely recognized as a marker for microglial cells; P2Y_12_ receptor expression is greatest on the ramified processes, and stimulation makes a major contribution to chemoattractant responses of microglial cells (Moore et al., 2015; Mildner et al., 2017). Stimulation of the adenosine A_2A_ receptor on microglial cells contributes to the characteristic amoeboid morphology during microglial activation accompanying brain inflammation (Orr et al., 2009).

The ionotropic P2X_7_ receptor is especially relevant to immune responses in microglial cells due to its requirement for high concentrations of ATP, its lack of inactivation, the distribution of particular splice variants amongst immune cells, the multiple interactions of its extended cytoplasmic tail, and its association with the opening of a large transmembrane pore (Di Virgilio et al., 2017). Receptor opening is stimulated by levels of ATP approaching the millimolar range, several orders of magnitude higher than required for other P2X or P2Y receptors (Coddou et al., 2011). While this was originally interpreted to mean the receptor was only activated by large levels of ATP released following cell rupture, the presence of the P2X_7_ receptor on several post-mitotic cells, combined with the identification of pannexin channels as ATP conduits localized adjacent to P2X_7_ receptors, provided for a role for the receptor in more widespread signaling scenarios (Miras-Portugal et al., 2017). Like other members of the P2X family, binding of ATP leads to the opening of a cation-selective channel that allows Ca^+^ and Na^+^ influx, and K^+^ efflux. However, in some circumstances stimulation also leads to the opening of a pore permeable to larger macromolecules up to 900 Da; whether this pore represents a more dilated state of the P2X_7_ channel or the recruitment of additional proteins such as pannexin channels remains unresolved, despite intense investigation (Di Virgilio et al., 2018). The lack of P2X_7_ channel inactivation in the continued presence of agonist has been attributed to the palmitoylated C-cys anchor retaining the gate in the open position (McCarthy et al., 2019). This lack of inactivation makes regulation of agonist levels critical for both initiating the signal and also shutting it down in a timely manner; there are parallels with the NMDA channel for glutamate in both the central role in signaling and the damage following overstimulation of both channels. The human P2X_7_ receptor possesses multiple splice variants, and the variant including the large 240 AA carboxy tail is usually associated with immune cells; this carboxy tail contains many of the binding sites associated with complex inflammatory interactions (Collo et al., 1997; Kaczmarek-Hajek et al., 2018; Kopp et al., 2019).

While the P2X_7_ receptor was originally referred to as “death receptor,” more recent work indicates the contribution of the receptor is much more nuanced, with participation in a variety of signaling events; stimulation can even lead to a proliferation in microglial cells (Bianco et al., 2006). These varied outcomes from the P2X_7_ receptor may be explained by differences in splice variant expression, agonist availability, and other factors still under investigation (Adinolfi et al., 2010; Di Virgilio, 2020). While excessive stimulation can lead to microglial cell death, the effects of the P2X_7_ receptor on the endolysosomal axis discussed here usually do not lead to cellular demise but have sustained pathological consequences for microglial clearance instead.

## Microglia, Inflammation, and Clearance

Microglia are morphologically plastic cells, existing in a variety of states in response to environmental stimuli. In the “resting” state, often referred to as M0, microglia engage in a constant sampling of their surroundings, with long ramified extensions surveying the neuronal milieu (Nimmerjahn et al., 2005). Microglia are constantly molded by environmental cues and respond to localized differences so that even in the resting state the population shows great heterogeneity (Lenz and Nelson, 2018). Once microglia are challenged with immunomodulatory substances, they enter an “activated” state in an attempt to maintain homeostasis. Although there is a range of “activated” states, they have traditionally been characterized into pro-inflammatory (“classical activation,” M1) and pro-phagocytic (“alternative activation,” M2) states (Colton, 2009). The M2 state itself can be subdivided into the M2a state associated with repair/regeneration functions, the M2b state associated with immunomodulatory functions, and the M2c state associated with an acquired deactivation phenotype (Mantovani et al., 2004; Chhor et al., 2013; Martinez and Gordon, 2014). Progression into these activation states follows clear triggers; application of lipopolysaccharide (LPS) or Interferon gamma (IFN-γ) leads to the M1-like states, application of Interleukin (IL) IL-4 or IL-13 triggers the M2a state, ligation of immunoglobulin Fc-gamma-receptors that results in IL-12 expression, increased IL-10 secretion, and HLA-DR expression leads to the M2b state, while IL-10 is associated with the M2c state (Akhmetzyanova et al., 2019). The states themselves are defined by proteomic or genetic markers, with IL-1β (*Il1b)* or NOS2/iNOS (*Nos2*) for M1, Arg1 (*Arg1*) or Chil3/YM1 (*Chil3*) for M2a, IL-10 (*Il10*) or CCL1 (*Ccl1*) for M2b and CD163 (*Cd163*), MMP8 (*Mmp8*), and VCAN (*Vcan*) for M2c (Stumpo et al., 2003; Mantovani et al., 2004; Boche et al., 2013; Chhor et al., 2013; Cherry et al., 2014; Martinez and Gordon, 2014; Lurier et al., 2017; Wang et al., 2019). However, recent investigations indicate this categorization is far too simplistic, and the different microglial states are better represented as a spectrum of combinations (Morganti et al., 2016; Lively and Schlichter, 2018). That said, the basic divisions between microglial states associated with increased inflammatory signals and those linked to increased phagocytosis are important to consider when interpreting the varied effects of the P2X_7_ receptor, and would benefit from a more thorough examination in the future.

The functional responses associated with the various microglial states usually help maintain the optimal activity of the neural tissue, a connection that can be overlooked when research is focused on pathology. However, the central role of microglia in coordinating the immune responses can lead to aberrant behavior upon excessive stimulation or impaired restraint. Microglia dysfunction has been implicated in several disease states, including schizophrenia (Tay et al., 2017), autism (Liao et al., 2020), ischemic stroke (Kanazawa et al., 2017), traumatic brain injury (Loane and Kumar, 2016), Alzheimer’s disease (Streit et al., 2020), Parkinson’s disease (Ferreira and Romero-Ramos, 2018) and frontotemporal dementia (Hickman et al., 2018). Understanding how to maintain the beneficial activities of microglia while limiting their destructive actions remains a key challenge, and the P2X_7_ receptor is likely to contribute to the balance with its central role in coordinating both inflammation and clearance.

The effects of the P2X_7_ receptor on microglial clearance are less well studied than those on inflammation, and throughout this review, results obtained from primary and cultured microglial cells are supplemented by work on macrophages and monocytes. While microglial cells share many features with their peripheral cousins the macrophages, including recognition through the widely used markers Iba1 or Cx3CR1, microglia differ from peripheral macrophages in several ways (Ransohoff and Perry, 2009; Li and Barres, 2018). For example, microglia differ from macrophages in protein expression (Butovsky et al., 2014; Bennett et al., 2016), turnover (Lawson et al., 1990; Askew et al., 2017), inflammatory response (Burm et al., 2016; Zarruk et al., 2018), and physiological regulation (Majumdar et al., 2007, 2008, 2011). Microglia originate from early myeloid progenitor cells that migrate from the yolk sac, with minimal CNS contribution from peripheral macrophages (Ginhoux et al., 2010; Gomez Perdiguero et al., 2015; Bennett et al., 2018). Of particular relevance here is the increased expression of degradative lysosomal enzymes such as dipeptidyl peptidase, tripeptidyl peptidase, and Cathepsin D (CatD) in microglial cells as compared to macrophages (Majumdar et al., 2007). Also important in this regard are differences between microglial cells from inflamed CNS and those recruited from blood-derived monocytes; the substantial differences recently identified between these cell types implies their response to the P2X_7_ receptor could well differ (Lapenna et al., 2018; Borst and Prinz, 2020; Yu et al., 2020). While the relative paucity of data from microglial cells on how the P2X_7_ receptor alters clearance means that some of the information presented in this review has been obtained from macrophages, the reader should be aware that application of results from macrophages to microglial cells has limitations.

## P2x_7_ Receptors and Microglial States

The P2X_7_ receptor is widely recognized for its inflammatory actions. Stimulation of the P2X_7_ receptor can result in the secretion of proinflammatory cytokines IL-1β and IL-6 in many cell types (Adinolfi et al., 2018; Burnstock and Knight, 2018; Shao et al., 2020). Additionally, stimulation of the P2X_7_ receptor leads to the nuclear translocation of transcription factor nuclear factor kappa-light-chain enhancer of activated B cells (NF-κB) which has been demonstrated to elevate gene expression of *Tnfa, Cox2, and Il1b* in microglia (Ferrari et al., 1997). While these markers are widely associated with the pro-inflammatory M1 state of microglia, the pattern of genes activated by stimulation of the P2X_7_ receptor in microglial cells is likely to be complex. M2 state markers Arg1 and CD163 occurred following 15-min stimulation with Benzoylbenzoyl-ATP (BzATP), a selective agonist of P2X_7_ (Fabbrizio et al., 2017). Together this may indicate a mixed activation state similar to what has been observed in microglia following traumatic brain injury (Morganti et al., 2016). Furthermore, isolated rat primary microglia challenged with LPS revealed upregulated expression of gene *Arg1* and its protein product (Lively and Schlichter, 2018). A direct comparison of LPS and IL-4 challenge to primary mouse microglia indicate that IL-4 induces an exponentially higher expression of *Arg1* compared to LPS (Chhor et al., 2013), however, suggesting the response is complex. Microglial activation state and autophagy may be interrelated, with autophagy induction polarizing microglia towards the M1 state, while induction of M2 state can itself increase autophagy (Fabbrizio et al., 2017).

## P2x_7_ Receptors and Phagocytosis by Microglial Cells

Phagocytosis of extracellular material is a central function of microglial cells. Materials bound to phagocytic receptors are taken in through the phagocytic cup and delivered to the endocytic system for pathogen destruction, degradation, or sorting (Solé-Domènech et al., 2016). Induction of the M2a state leads to an increase in cell-specific receptors associated with phagocytosis such as CD163, TREM2, and Dectin-1 (Willment et al., 2003; Neumann and Takahashi, 2007; Kowal et al., 2011; Zhang et al., 2012; Cui et al., 2020; Kim et al., 2020). However, the expression of phagocytosis receptors does not necessarily correlate with increased phagocytosis, and the relationship between induction into the M2a state and the rate of phagocytosis is a matter of debate (Márquez-Ropero et al., 2020). Induction of both M1 and M2 microglial phenotypes was found to reduce deposits of amyloid-beta *in vivo* (Tang and Le, 2016), while the addition of IL-4 suppressed macrophage phagocytosis of beads *in vitro* (Moreno et al., 2007). Stimulation of receptors such as TREM2 and MerTK modulate microglial phagocytosis (Nomura et al., 2017; Janda et al., 2018), but the relationship between the P2X_7_ receptor and microglial phagocytosis by microglia are more complex. For example, P2X_7_ receptor expression was not upregulated with the challenge by lipopolysaccharides (Raouf et al., 2007), but was upregulated with exposure to amyloid-beta (1–42; McLarnon et al., 2006), suggesting P2X_7_ expression is a specific response.

**Figure 1:**
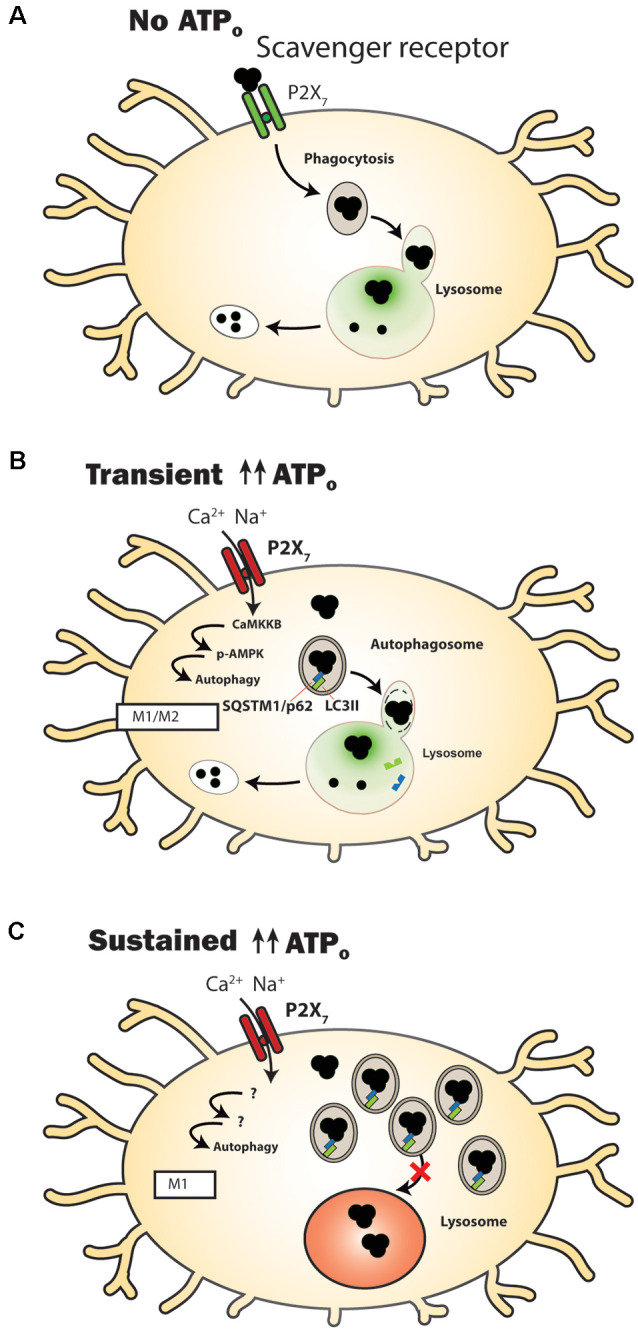
P2X_7_ receptor modulation of clearance pathways in microglial cells. **(A)** Under control conditions when extracellular adenosine triphosphate (ATP) levels (ATP_o_) are too low to activate the P2X_7_ receptor, it acts as a scavenger receptor to aid in the phagocytosis of extracellular waste. The luminal pH of lysosomes in microglial cells is sufficiently acidic (green) to enable efficient degradative enzyme activity. **(B)** Following transient stimulation with high concentrations of ATP_o_, the P2X_7_ receptor no longer acts as a scavenger receptor. Autophagy is stimulated, shown by a transient rise in levels of autophagosomal lipid marker microtubule-associated protein 1A/1B-light chain 3-II (LC3-II). Material is degraded and microglia express genes and proteins associated with a mixed M1/M2 activation state. **(C)** A more prolonged stimulation of the P2X_7_ receptor leads to an elevated lysosome pH (red), with autophagosomal contents being delivered extracellularly instead of to the lysosome. Markers of accumulation, including LC3-II (green) and sequestosome-1 (SQSTM1/p62; blue) are elevated. Microglia express genes and proteins associated with an M1 activation state.

One of the most intriguing connections between the P2X_7_ receptor and phagocytosis involves its identity like a scavenger receptor, whereby the receptor increases phagocytosis in the absence of external agonist ATP (Gu et al., 2010, 2011; Gu and Wiley, 2018; Figure 1A). This role of the P2X_7_ receptor differs considerably from other functions in that activity as a scavenger receptor is reduced when extracellular ATP rises. For example, phagocytic uptake of non-opsonized beads by cells of the human monocyte THP-1 cell line was reduced by blocking the receptor with antibodies against the extracellular domain of the P2X_7_ receptor (Gu et al., 2010). A similar reduction in phagocytosis of *E. coli* bacteria by human-derived monocytes was also induced by a P2X_7_ receptor antibody (Ou et al., 2018). Pharmacological support for the P2X_7_ receptor contribution was shown in human microglial cells when stimulation of the P2X_7_ receptor with agonist BzATP reduced phagocytosis of fluorescent-tagged *E. coli* bioparticles, while P2X_7_ antagonist A438079 reversed the effect of BzATP and enhanced phagocytosis of these bioparticles (Janks et al., 2018). Further support for a role for the unstimulated P2X_7_ receptor in phagocytosis was shown on a molecular level in experiments where siRNA knockdown of the P2X_7_ receptor reduced phagocytosis in the absence of agonist in isolated human monocytes, while overexpression of the P2X_7_ receptor in HEK293 cells increased phagocytosis (Gu et al., 2010, 2011).

Details about the mechanisms of P2X_7_ as a scavenger receptor show a complex pattern. Extracellular residues 306–320 of the P2X_7_ receptor were shown to bind to beads, bacteria, and apoptotic cells (Gu et al., 2011). Intracellular regions of the P2X_7_ receptor are bound to non-muscle myosin heavy chain IIA (NMMHC IIA) and thus the cytoskeleton (Gu et al., 2009, 2010), consistent with a pathway for phagocytosis of material into the cell. The addition of ATP led to dissociation of NMMHC IIA from the P2X_7_ receptor and reduced phagocytosis with roughly the same kinetics as channel gating, suggesting a common link. Overexpression of NMMHC IIA reduced the pore-opening activity that occurred with P2X_7_ receptor agonist presentation, reinforcing the notion that the receptor may switch between its phagocytic and conductive identities (Gu et al., 2009). However, P2X_7_ receptor-selective antagonists blocked pore formation but not phagocytic activity in human monocytes (Ou et al., 2018), indicating that phagocytosis is driven by the closed state of the receptor, and antagonist binding sites are distinct from the phagocytic targets (Gu and Wiley, 2018). This suggests these P2X_7_ antagonists can block channel activity without affecting phagocytosis, a useful selection trait if the goal is to reduce the balance between inflammatory activity and phagocytosis (Fletcher et al., 2019). Transient stimulation of the P2X_7_ receptor in human-derived microglia decoupled the receptor from the cytoskeleton and eliminated its ability to phagocytose bioparticles while also activating caspase-1 (Gu et al., 2009, 2010). Overall, the ability of the P2X_7_ receptor to enhance phagocytosis in the absence of extracellular ATP presents a critical step in understanding how the receptor manipulates microglial clearance of extracellular debris. Moreover, P2X_7_ receptor phagocytic activity is likely restricted to microglia, as phagocytosis by human monocyte-derived macrophages was inhibited with a glycoprotein-rich fraction of serum (Gu et al., 2012). Readers are directed to the excellent review by Gu and Wiley for more details about the P2X_7_ receptor as a scavenger receptor (Gu and Wiley, 2018).

While the ability of the P2X_7_ receptor to act as a scavenger receptor in the absence of an agonist is strongly supported by the evidence above, it should be noted that not all experimental findings agree with this concept. For example, recent work in murine bone marrow-derived macrophages (BMDMs) suggests that stimulation of the P2X_7_ receptor enhances uptake of pHRodo-tagged bacterial bioparticles, as uptake was reduced following exposure to P2X_7_ receptor inhibitor A740003 and by reduction of extracellular ATP with the hydrolyzing enzyme apyrase (Zumerle et al., 2019); phagocytosis was not inhibited with antagonists AZ10606120 and A438079, however (Allsopp et al., 2018). Whether these differences indicate a characteristic of BMDMs or just experimental deviation remains to be determined.

## P2x_7_ Receptors and Regulation of Lysosome Ph and Autophagosome Maturation

Lysosomes are membrane-bound organelles with an acidic lumen that are responsible for the degradation of extracellular materials delivered through the endocytic pathway, or of intracellular materials delivered *via* autophagy. Degradation is accomplished by several intraluminal hydrolases, whose enzymatic activity is optimally at low pH levels (Stoka et al., 2016). In addition to being necessary for optimal degradative activity, a low lysosomal pH is important for maintaining the electrical potential across the lysosomal membrane, the transport of signaling molecules stored in lysosomes such as Ca^2+^ and ATP, and for the fusion of the lysosomal membrane with incoming autophagosomes and endosomes (Xu and Ren, 2015; Perera and Zoncu, 2016). Elevation of lysosomal pH reduces degradation of phagocytosed material in numerous cell types (Sarkar et al., 2009) and leads to autophagic backup and reduced protein degradation (Klionsky et al., 2021), making the restoration of an acidic lysosomal pH a key target for the amelioration of diseases of accumulation (Guha et al., 2014).

The ability of P2X_7_ receptor stimulation to elevate lysosomal pH in numerous cell types including microglia has important implications for clearance (Figures 1B,C). The P2X_7_ receptor deacidified lysosomes in microglial cells; stimulation of the MG6 microglia cell line with ATP (Takenouchi et al., 2009) or the BV2 microglial cell line with P2X_7_ receptor agonist BzATP (Sekar et al., 2018) elevated lysosomal pH. The ability of P2X_7_ receptor antagonists to prevent this de-acidification supported a role for the P2X_7_ receptor in this rise in lysosomal pH. It should be noted, however, that P2X_7_ receptor stimulation of macrophages infected with bacteria acidified lysophagosomes (Fairbairn et al., 2001).

The relatively high lysosomal pH of microglial cells under baseline conditions differs from peripheral macrophages and other cells, with a reported value of 6.0 as compared to 4.5–5 in most cell types (Majumdar et al., 2007). However, the lysosomal pH in microglia is dynamic, and several pathways are involved in lowering the luminal pH in preparation for degradative activity. For example, stimulation with microglial activating compound Macrophage Colony Stimulating Factor (MCSF) or LPS lowered lysosomal pH and enhanced degradation of protofibrillar amyloid-beta (fAβ; Majumdar et al., 2007). MCSF putatively polarizes microglia to the M2 state while LPS polarizes them towards the M1 state (Cherry et al., 2014; Hu et al., 2015). This study also demonstrated that forskolin lowered lysosomal pH and increased fAβ degradation, and the enhanced degradation induced by MCSF was blocked by inhibition of protein kinase A or chloride transport, consistent with cyclic adenosine monophosphate (cAMP) and chloride contributing to lysosomal acidification. The movement of chloride transporter CLC-7 into the lysosomal membrane to enact Cl^−^ counterion transport was subsequently identified as a key mechanistic step in luminal acidification (Majumdar et al., 2011). Future experiments are needed to determine whether baseline lysosomal pH differs in microglia of the pro-phagocytic M2 state and whether the rise in lysosomal pH associated with P2X_7_ receptor stimulation is altered in this subset of cells.

**Figure 2:**
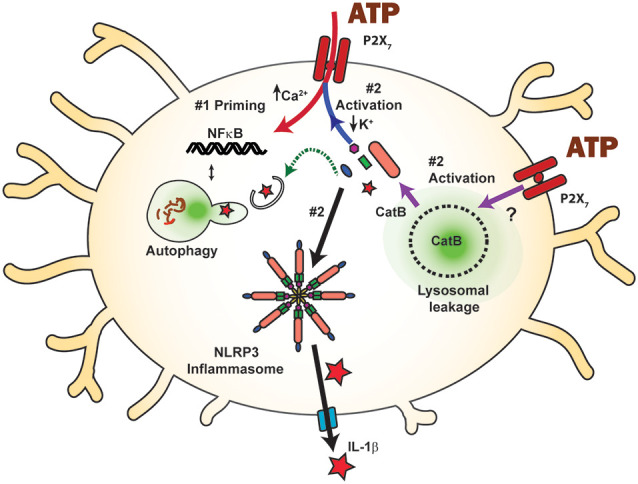
Inflammasome activation by the P2X_7_ receptor and the balance between priming and autophagic degradation of inflammasome components. Stimulation of the P2X_7_ receptor triggers the assembly and activation (step #2) of the NLRP3 inflammasome following efflux of K^+^ through the open channel (blue arrow), eventually leading to cleavage of pro-IL-1β (red star) into the mature form and release through the gasdermin D pore (blue). The P2X_7_ receptor can also activate the NLRP3 inflammasome and subsequent IL-1β release by raising cytoplasmic cathepsin B (CatB), following its initiation of lysosomal leakage through an unknown mechanism (purple arrows). P2X_7_ receptor stimulation can prime inflammasome components *via* NF-κB (step #1, red arrow) and increase available inflammasome components. Component levels can also be decreased following autophagic degradation (green arrow), limiting the inflammatory potential of P2X_7_ receptor stimulation. The balance between priming and autophagic degradation will influence the inflammatory impact of P2X_7_ receptor stimulation.

A rise in lysosomal pH will usually reduce rates of degradation or autophagy. After extracellular debris is engulfed into the cell *via* phagocytosis, it moves through a membrane enclosure called an autophagosome, which eventually binds to and fuses with the lysosome for hydrolase-mediated degradation. There are suggestions that stimulation of the P2X_7_ receptor may reduce autophagosome-lysosome fusion to further reduce bacterial clearance. ATP reduced the fluorescence signal in MG6 microglial cells that had phagocytosed pHrodo-BioParticles (Takenouchi et al., 2009); as pHrodo-BioParticles fluoresce brighter in the lower pH levels of lysosomes, this implied a reduced delivery to the lysosomal lumen, although this is also consistent with a de-acidification of that compartment. Furthermore, exposure to 2.5 mM ATP for 4 h resulted in detection of the transferrin receptor protein, the intermediate form of endolysosomal protease CatD and autophagosomal lipid marker microtubule-associated protein 1A/1B-light chain 3-II (LC3-II) into the supernatant, consistent with incomplete autophagosome-lysosome maturation and elevated secretion into the extracellular space (Takenouchi et al., 2009) *via* a non-classical secretory pathway (Dubyak, 2012). Lysosome destabilization can also lead to the release of lysosomal cathepsins into the cytoplasm (Nakanishi, 2020). Intracellular cathepsin B (CatB) is known to promote cleavage of cytokine IL-1β *via* caspase one after stimulation of the P2X_7_ receptor (Figure 2). Furthermore, CatB activity can polarize microglia closer to M1, with microglia isolated from hippocampi after hypoxia/ischemia expressing significantly higher levels of *Nos2, Tnfa, and IL1b* not observed in those from CatB^−/−^ mice (Ni et al., 2015).

The above contrasts with *in vitro* studies of human and mouse macrophages which suggest that the P2X_7_ receptor can enhance phagosome-lysosome fusion and increases degradation of bacteria in a calcium and phospholipase D (PLD)-dependent manner (Kusner and Barton, 2001; Stober et al., 2001; Coutinho-Silva et al., 2009; Corrêa et al., 2010). Mouse macrophages that internalized *Mycobacterium bovis* Bacille Calmette-Guérin (BCG) followed by exposure to 3 mM ATP for 4 h showed increased colocalization between BCG and lysosome marking-dye Lysotracker Red (Fairbairn et al., 2001). P2X_7_ receptor stimulation reduced the viability of *Mycobacterium tuberculosis* in primary human macrophages and THP-1 derived macrophages, dependent upon Ca^2+^ influx and activation of PLD (Kusner and Adams, 2000; Kusner and Barton, 2001). Inhibition of PLD was independent of Ca^2+^ influx and correlated with the reduction of phagosome-lysosome fusion. However, internal calcium stores were involved with the loss of internalized *M. Bovis* that were associated with high levels of extracellular ATP (Stober et al., 2001). These differences between effects of the P2X_7_ receptor on phagocytosis by macrophages vs. microglia again stress the need for further examination of microglia alone.

## Manipulation of Autophagy by The P2x_7_ Receptor

Autophagy is the process by which intracellular material is identified, packaged, and trafficked for lysosomal degradation, and several lines of evidence suggest the P2X_7_ receptor can modulate the autophagy process (Figure 1). Autophagy is normally prevented by the mammalian target of rapamycin 1 (mTOR1); inhibition of mTOR by AMP-activated protein kinase (AMPK) can thus initiate autophagy (Nazio et al., 2013; Hindupur et al., 2015). Recruitment of LC3 to membranes leads to the formation of nascent autophagosomes, and increased clustering of the membrane-bound phosphatidylethanolamine-conjugated LC3-II is a common marker for autophagy initiation (Klionsky et al., 2021). Autophagy, by definition, focuses on processing material produced within microglial cells, but autophagic and phagocytic pathways run in parallel. While autophagy (Plaza-Zabala et al., 2017), phagocytosis (Sierra et al., 2013; Solé-Domènech et al., 2016), and their intersection with inflammation (Su et al., 2016) within microglia have been individually reviewed, the specific effects of P2X_7_ receptor stimulation on the autophagic pathway in microglia merit special attention.

Transient stimulation of the P2X_7_ receptor with ATP or BzATP elevates LC3-II levels in isolated primary mouse microglia, with LC3-II returning to basal levels 4 h after receptor stimulation (Takenouchi et al., 2009). A similar rise in LC3 was found in the MG6 (Takenouchi et al., 2009) and BV2 (Sekar et al., 2018) microglial cell lines. Receptor involvement is supported by a reduced LC3-II response to stimulation in microglial cells from P2X_7_^−/−^ mice and in the presence of P2X_7_ antagonists. The rise in LC3-II was greater in microglial cells from the SOD1-G93A mouse model of amyotrophic lateral sclerosis (ALS) than the wild type, and peaked 15 min after agonist presentation (Fabbrizio et al., 2017).

Elevation of LC3-II can represent autophagic induction or autophagic backup (Klionsky et al., 2021); the ability of the P2X_7_ receptor to elevate lysosomal pH, combined with the autophagic reduction that accompanies lysosomal de-acidification (Kawai et al., 2007), demands particular care when investigating how the P2X_7_ receptor increases LC3-II levels. Levels of LC3-II were increased further when proton pump inhibitor Bafilomycin A1 was given concurrently with BzATP (Fabbrizio et al., 2017); while this is a classical test to distinguish between autophagy induction and inhibition, the relatively small increase, combined with the absence of absolute pH measurements, does not rule out an effect of lysosomal de-acidification on rising LC3-II levels. The BzATP-mediated rise in LC3-II was dependent upon extracellular calcium in microglia and THP monocytes (Biswas et al., 2008; Takenouchi et al., 2009), but extracellular calcium was also necessary for lysosomal deacidification (Guha et al., 2013). However, additional observations support the ability of the P2X_7_ receptor to raise LC3-II by inducing autophagy, independent of its effects on lysosomal pH. In microglia derived from the superoxide dismutase 1 (SOD1)-G93A mouse model, sequestosome-1 SQSTM1/p62 levels were reduced by 15 min BzATP but increased by a sustained stimulation with BzATP for 6 h (Fabbrizio et al., 2017). As SQSTM1/p62 normally binds to cargo targeted for autophagic degradation by the lysosome and is itself degraded, p62 accumulation is a standard indicator of autophagic backup due to a reduced lysosomal function (Klionsky et al., 2021). However, SQSTM1/p62 is a multifunctional protein implicated in several cellular interactions such as adipogenesis, nutrient sensing, oxidative stress reactions though nuclear factor erythroid 2-related factor (Nrf2), and regulation of NF-κB. Additionally, both Nrf2 and NF-κB have transcriptionally upregulated expression of SQSTM1/p62 (Puissant et al., 2012; Sánchez-Martín et al., 2019). As such, the complex time course linking the P2X_7_ receptor to p62 elevation may reflect involvement of pathways in addition to degradation.

The activation of the AMPK pathway following stimulation of P2X_7_ receptors also provides support for autophagic induction by the P2X_7_ receptor, given the role of AMPK in regulating autophagy induction (Kim et al., 2011). For example, BV2 cells stimulated with BzATP showed an elevation of p-AMPK, consistent with autophagy induction (Sekar et al., 2018). LC3-II protein levels were reduced with siRNA against AMPK, AMPK inhibition with Compound C, or the addition of mitochondrial antioxidant MitoTEMPO. The activation of AMPK by calcium-sensitive calcium/calmodulin-dependent protein kinase beta (CaMKKβ; Bootman et al., 2018) may link calcium influx through the P2X_7_ channel and AMPK activation. The demonstration of rapid phosphorylation of mTOR after BzATP application also supports autophagy induction downstream from AMPK-activation (Fabbrizio et al., 2017). Whether P2X_7_ receptor signaling leads to canonical inhibition of mTOR, activation of AMPK or additional pathways requires further clarification. Future investigations must control for the effect of P2X_7_ receptors on lysosomal pH, and the considerable feedback systems mediated by Transcription factor EB (TFEB) that are activated by this rise in pH (Zhitomirsky et al., 2018). However, the identification of intersections between the P2X_7_ receptor and autophagy pathways is an active field of investigation in microglial research and multiple targets should soon emerge.

## Effect of Priming and Autophagy on P2x_7_ Receptor-Mediated Inflammatory Responses

While this review is focused on how the P2X_7_ receptor modifies microglial clearance, autophagic clearance can also modulate the impact of the P2X_7_ receptor on inflammation. The P2X_7_ receptor is well known for its association with increased inflammatory signals and cytokine release (Figure 2); its interaction with the NLRP3 inflammasome is perhaps the most widely recognized (Di Virgilio et al., 2017), and the P2X_7_ receptor was the most potent activator of the NLRP3 inflammasome in N13 microglial cells (Franceschini et al., 2015). The NLRP3 inflammasome is a multimeric compound consisting of NOD-, LRR- and pyrin domain-containing protein 3, the apoptosis-associated speck-like protein containing caspase recruitment domain (ASC), and procaspase-1 (Swanson et al., 2019). The resulting release of master cytokine IL-1β can influence many immune signals, and the inflammasome is tightly regulated to reflect its central role. In the first “priming” step, the availability of inflammasome components is controlled. In the second step, these components assemble to form the inflammasome itself, resulting in sequential cleavage of caspase-1 and then cytokines IL-1β and IL-18 into their mature forms. These mature cytokines exit the cell through the cleaved gasdermin D pore, enabling cytokine release into extracellular space where they initiate a variety of inflammatory responses. The efflux of K^+^ from the cell is a key signal to assemble component proteins into the inflammasome, and this efflux is frequently associated with the opening of the P2X_7_ receptor (Muñoz-Planillo et al., 2013; Katsnelson et al., 2015; Swanson et al., 2019).

While the P2X_7_ receptor is closely associated with the secondary activation stage of the NLRP3 inflammasome, reports also link it to the first priming phase. Priming is often initiated experimentally with the Toll-Like Receptor 4 agonist LPS, which leads to translocation of transcription factor NF-κB to the nucleus, resulting in the transcription of inflammasome components (Kahlenberg et al., 2005). However, several studies suggest that the P2X_7_ receptor can also fulfill the priming step, initiating translocation and IL-1β transcription itself. N9 mouse microglia cells exposed to ATP or BzATP demonstrated NF-κB activation, though the response was delayed when compared to LPS-mediated activation (Ferrari et al., 1997). This study also demonstrated that the NF-κB/DNA complex induced by P2X_7_ receptor signaling differed from that induced by LPS, in that ATP induced a p65 homodimer, rather than the p65/p50 heterodimers found with LPS. Work in HEK293T cells and the RAW264.7 macrophage cell line indicate that the P2X_7_ receptor also interacts with myeloid differentiation primary-response protein 88 (MyD88), a common adaptor protein for TLRs (Liu et al., 2011). P2X_7_ receptor-induced NF-κB translocation has been demonstrated in osteoclasts (Korcok et al., 2004) and osteoblasts (Genetos et al., 2011), suggesting that P2X_7_ receptor activation of NF-κB may be a common mechanism for priming inflammasome components. In astrocytes, data showing inflammasome priming of isolated cells by the P2X_7_ receptor was supplemented by a role *in vivo*, in which increased expression of IL-1β followed injection of BzATP and the pressure-dependent rise in IL-1β was absent in P2X_7_^−/−^ mice (Albalawi et al., 2017). This ability of P2X_7_ receptor activation to both prime and activate the NLRP3 inflammasome places it in a very powerful position to regulate innate immune responses.

Numerous studies highlight interactions between autophagy and the NLRP3 inflammasome (Biasizzo and Kopitar-Jerala, 2020); while the specific role of the P2X_7_ receptor is usually not involved, autophagic degradation of inflammasome components is predicted to regulate the ability of the P2X_7_ receptor to activate the NLRP3 inflammasome. Increased autophagy is associated with decreased NLRP3 inflammasome function *in vivo* in rodent microglial cells (Chen et al., 2019), and in primary microglia cells (Cho et al., 2014; Chen et al., 2019; Han et al., 2019). Autophagic degradation of inflammasome components was also suggested by studies in the BV2 cell line (Shao et al., 2014; Kim et al., 2015; You et al., 2017; Han et al., 2019), in THP-1 human macrophages (Liu et al., 2018), and in BMDCs or immortalized bone marrow-derived macrophages (Harris et al., 2011; Sun et al., 2017). Induction of autophagy by a small molecule reduced NLRP3 expression in microglial cells and inflammasome activation (Han et al., 2019). Suppressed caspase-1 activation may lead to enhanced autophagy in microglial cells following inflammasome silencing (Lai et al., 2018). Primary microglia derived from mice deficient in autophagy gene Beclin-1 (BECN^−/−^) had less colocalization between NLRP3 and LC3 than their wild-type counterparts, and increased IL-1β levels, suggesting that autophagic degradation can reduce the NLRP3 inflammasome response (Houtman et al., 2019). Colocalization between ASC or NLRP3 and LAMP-1 after induction of autophagy was demonstrated in THP-1 cells (Shi et al., 2012). Similarly, IL-1β colocalized with LC3-GFP puncta in immortalized BMDMs (Harris et al., 2011). Deficits in autophagy and clearance are predicted to promote P2X_7_-mediated inflammation, as the accumulation of lipid droplets within microglia resulted in reduced phagocytosis and increased secretion of proinflammatory cytokines IL-1β and Tnfα after the LPS challenge (Marschallinger et al., 2020). In BMDMs, autophagy induction increased IL-1β secretion dependent upon ATG5 (Dupont et al., 2011). However, degradation of inflammasome components through the autophagy pathway is generally expected to reduce the ability of the P2X_7_ receptor to initiate assembly and activation of the inflammasome in microglial cells. Overall, the balance between the degradation of inflammasome proteins and their upregulation *via* priming initiated by P2X_7_ receptor stimulation will influence the consequences of NLRP3 inflammasome activation by the P2X_7_ receptor on microglial cells (Figure 2).

## The P2x_7_ Receptor Activates The Nlrp3 Inflammasome *Via* Lysosomal Leakage and Cathepsin B

**Figure 3:**
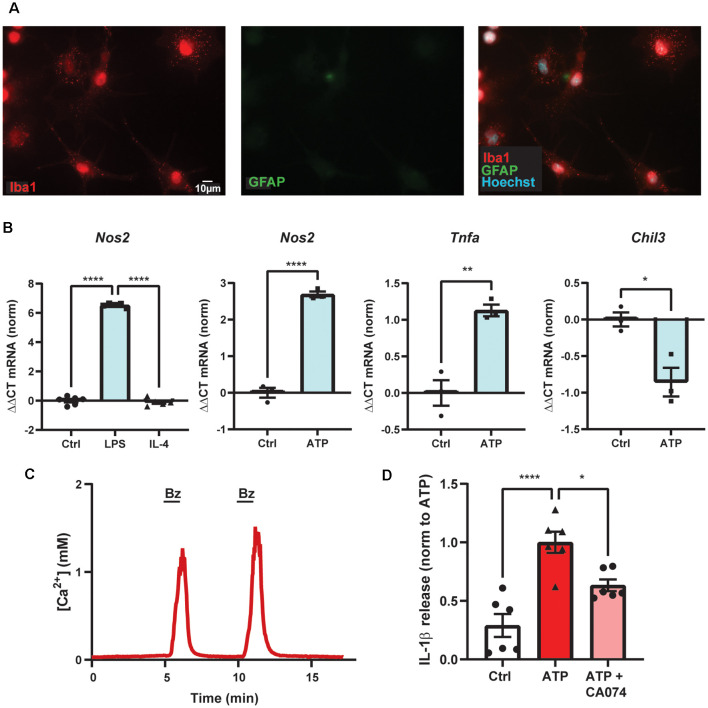
ATP-dependent IL-1β release from primary microglial cells involves cathepsin B. **(A)** Purity of primary mouse brain microglia cultures shown with high staining for marker Iba1 (red) and low staining for astrocytic marker GFAP (green). **(B)** Primary microglial cultures increased expression of *Nos2* with 4 h exposure of LPS but not IL-4, as expected for microglial cells polarized into the M1-state (****p < 0.001). Exposure to 1 mM ATP for 4 h increased expression of *Nos2* (****p < 0.001) and *Tnfa* (**p = 0.0042) and decreased expression of *Chil3* (*p = 0.014) as compared to control (Ctrl) using quantitative PCR. **(C)** Evidence of functional P2X_7_ receptors in primary murine microglial cells indicated by the robust, reversible and repeatable elevation of cytoplasmic calcium when briefly exposed to 100 μM BzATP (line); cells were loaded with Ca^2+^ indicator fura-2. **(D)** Release of cytokine IL-1β triggered by exposure to 1 mM ATP for 1 h was reduced by cathepsin B inhibitor CA-074 (10 μM). Microglial cells were primed with 500 ng/ml LPS for 4 h and IL-1β was measured with an ELISA. Approaches as described previously (Lim et al., 2016; Lu et al., 2017; Shao et al., 2020). All work was approved by the University of Pennsylvania IACUC.

A particularly important connection linking the P2X_7_ receptor, lysosomes, and inflammation involves the ability of the P2X_7_ receptor to cause lysosomal destabilization and subsequent activation of the NLRP3 inflammasome through actions of cathepsin B. In BMDMs, destabilized lysosomes led cathepsin B to activate caspase-1 and cleave IL-1β for release (Chevriaux et al., 2020). In the THP-1 monocytic cell line, *S. pneumoniae* triggered IL-1β release in an ATP- and cathepsin B-dependent manner (Hoegen et al., 2011). In BV-2 cells, increased leakage of cathepsin B into the cytoplasm following BzATP application was shown using the Magic Red assay, while inhibition of cathepsin B with CA-074 prevented the BzATP-induced cell death (Sekar et al., 2018). In our studies on primary mouse brain microglial cells, the ATP-dependent secretion of IL-1β was reduced by cathepsin B inhibitor CA-074 (Figure 3). While these findings suggest a critical mechanism by which the P2X_7_ receptor can activate the NLRP3 inflammasome by disrupting lysosomal integrity, additional work is needed to understand how receptor activation leads to lysosomal leakage and whether this is related to its ability to increase lysosomal pH. Experiments to determine whether compromised lysosomes filled with partially degraded waste material are more susceptible to this lysosomal leakage are warranted.

## Discussion

This review article has presented emerging evidence for the role of the P2X_7_ receptor in regulating clearance by microglial cells. While the contributions of the P2X_7_ receptor to immune signaling are well known (Orioli et al., 2017; Adinolfi et al., 2018), its ability to modulate microglial clearance has been less well studied. The ability of the P2X_7_ receptor to mediate clearance of extracellular and intracellular material occurs at several key steps including the unstimulated receptor acting as a scavenger receptor to phagocytose extracellular material into the microglial cells, the transient receptor stimulation enhancing autophagy through a p-AMPK pathway, and the sustained receptor stimulation elevating lysosomal pH and reducing degradation. Given its role at the crossroads between inflammation and clearance, the ability of the P2X_7_ receptor to activate the NLRP3 inflammasome following lysosomal leakage is particularly relevant. Also, regulating the availability of inflammasome components by balancing their decrease through autophagy with their increase through priming has special relevance as the P2X_7_ receptor can itself increase priming. We propose the effects of the P2X_7_ receptor on clearance pathways in microglia are as significant as its effects on inflammatory signaling. Given that impaired clearance by microglial cells is increasingly implicated in the pathogenesis of neurodegenerative diseases (Bachiller et al., 2018), regulation of this clearance by the P2X_7_ receptor warrants further investigation, and identifies potential points of intervention.

Given the receptor involvement in both clearance and inflammation, several points of relevance emerge. First, the ability of the P2X_7_ receptor to act as a scavenger receptor offers several new directions for therapeutics (Gu and Wiley, 2018; Fletcher et al., 2019). Because this role requires the absence of an agonist, reducing extracellular ATP levels may provide the double benefit of increasing phagocytosis while reducing inflammation. Overexpression of intracellular binding protein NMMHC IIA reduced P2X_7_ receptor pore opening, suggesting that shifting the receptor towards this phagocytic role may also reduce its cytotoxic actions.

Second, the relatively high baseline level of pH in the lumen of microglial lysosomes implies that regulation of this pH is a powerful mechanism through which external and internal signals can increase clearance. Given that stimulation of the P2X_7_ receptor raises lysosomal pH, pH may be lowered by receptor antagonists, or by reducing levels of agonist ATP by increasing enzymatic degradation of extracellular ATP and blocking ATP release through sites like pannexins. Elevation of cAMP and chloride channel function lowered lysosomal pH in microglial cells (Majumdar et al., 2007, 2011), identifying a potential pathway to enhance clearance. Our work indicates the elevation of cAMP and PKA can acidify compromised lysosomes in epithelial cells (Liu et al., 2008), and that stimulation of plasma membrane receptors linked to stimulatory G proteins, such as the dopamine D5 receptor, can lower lysosomal pH and increase the clearance of phagocytosed waste by impaired lysosomes (Guha et al., 2012). Increased clearance can also be induced by stimulating the cystic-fibrosis transmembrane conductance regulator (CFTR) chloride channel localized to the lysosomal membrane (Liu et al., 2012), or by acid nanoparticles transported to the lysosomal lumen (Baltazar et al., 2012; Lööv et al., 2015). Antagonists targeted to the P2Y_12_ receptor were recently shown to lower lysosomal pH and improve clearance; of note, oral delivery of the FDA-approved antagonist ticagrelor (Brilinta) lowered lysosomal pH and was protective in a mouse model of neurodegeneration (Lu et al., 2018, 2019). Whether this drug can cross the blood-brain barrier to reach microglial cells, and whether it will interfere with other microglial functions regulated by the P2Y_12_ receptor remains to be determined. However, these studies provide a proof of concept for pharmacologic restoration of an acidic lysosomal lumen to enhance clearance.

Third, the ability of the P2X_7_ receptor to activate the NLRP3 inflammasome following lysosomal leakage of cathepsin B identifies another pathway through which limiting P2X_7_ receptor stimulation may be of benefit. Given that lysosomal accumulations and inflammatory signaling are both emerging as key steps in aging-dependent neurodegenerations, it would be interesting to see whether leakage of cathepsin B occurs more frequently from compromised lysosomes and whether this becomes a dominant pathway to NLRP3 inflammasome activation in aging microglial cells.

Finally, manipulating the balance between increasing levels of inflammasome components through priming and decreasing levels following their autophagic removal will have a considerable impact on the immune signals emerging from the microglial cell. In this regard, the ability of the P2X_7_ receptor to trigger both the stage one “priming” of the inflammasome as well as the stage two “activation” phase suggests the P2X_7_ receptor, and its agonist extracellular ATP, have important consequences for the neuroinflammatory environment. The autophagic degradation of inflammasome components represents an “anti-priming” and emphasizes how the ratio of increasing and decreasing relevant inflammasome proteins can control the gain on the inflammasome responses triggered by the P2X_7_ receptor, including those activated by lysosomal leakage.

While some therapeutic options of P2X_7_ receptor inhibition have been explored (Burnstock and Knight, 2018) a deeper understanding of the intersection between the P2X_7_ receptor, clearance, and inflammation will assist treatments for neurodegenerative diseases in which both problems present. In age-related macular degeneration, where increased accumulations and loss of microglia clearance occur, shifting the P2X_7_ receptor away from its inflammatory contributions and towards its scavenging role may prove advantageous (Vessey et al., 2017; Fletcher et al., 2019). In Alzheimer’s disease, aged microglia incompletely digest amyloid-beta plaques, increasing local inflammation and pathogenesis (Heneka et al., 2015; Streit et al., 2020). The detrimental effects of Rapamycin to neurons in preclinical models of Alzheimer’s disease after plaque deposition (when clinical diagnosis would likely occur) illustrate the complexity of manipulating a system as tightly regulated as autophagy (Carosi and Sargeant, 2019; Kaeberlein and Galvan, 2019), and stress the need for better understanding and a more nuanced approach. Finding therapeutic modalities that ameliorate autophagic reduction, or ones that can be targeted away from neurons, is crucial. In conditions of trauma such as spinal cord injury that are accompanied by elevated ATP and autophagic dysregulation (Galluzzi et al., 2016; Li et al., 2019), the modulation of microglial activation state has been proposed to enhance repair (Hu et al., 2015; Akhmetzyanova et al., 2019; Kobashi et al., 2020). However, treatment timing is critical, as transient P2X_7_ receptor stimulation may enhance autophagy and promote M2a microglia, while sustained stimulation can inhibit autophagy and promote proinflammatory M1 microglia (Fabbrizio et al., 2017; Jiang et al., 2021). The P2X_7_ receptor plays a complex role in microglial cells in balancing clearance with inflammatory signals; deeper mechanistic investigations may reveal more specific therapeutic approaches.

The above observations come with multiple caveats, and there remain some inconsistencies. For example, microglial responses differ from those in macrophages on several levels, such as the effect on phagosome-lysosome fusion. As many of the findings come from *in vitro* experiments, even results obtained from “microglial” cells can vary with the use of cell lines or primary culture. *In vitro* microglia have been demonstrated to lose transcriptional identity in as little as 8 days after isolation (Bennett et al., 2018). With a cell type so sensitive to extracellular environments, even small changes in the protocol can alter responsiveness. Also, microglial cells *in vitro* are removed from the challenges of the neurodegenerative environment, and their need to phagocytose waste material, along with the presence of accumulating waste material, will be altered significantly. Closer attention to the effect of the microglial state on responses to the P2X_7_ receptor is also needed.

In summary, the P2X_7_ receptor can modulate both inflammatory and clearance pathways in microglial cells. Reducing extracellular ATP levels to shift the receptor from an inflammatory trigger to a scavenger receptor will likely be of benefit in neuroinflammatory diseases associated with increased waste material. Limiting conditions in which receptor stimulation leads to lysosomal leakage will also help reduce NLRP3 inflammasome activation. The P2X_7_ receptor makes a complex contribution to microglial function, and greater attention in future experiments to attributes such as microglial polarization and lysosomal waste accumulation will help clarify these interactions.

## Author Contributions

CM and KC contributed to the design, writing and editing of this manuscript. All authors contributed to the article and approved the submitted version.

## Conflict of Interest

The authors declare that the research was conducted in the absence of any commercial or financial relationships that could be construed as a potential conflict of interest.
